# Guidelines for Myelodysplastic Syndromes: Converting Evidence into Action?

**DOI:** 10.3390/ijerph18147629

**Published:** 2021-07-18

**Authors:** Annika Kasprzak, Jennifer Kaivers, Kathrin Nachtkamp, Rainer Haas, Guido Kobbe, Norbert Gattermann, Ulrich Germing

**Affiliations:** Department of Hematology, Oncology and Clinical Immunology, Heinrich-Heine-University, 40225 Düsseldorf, Germany; Jennifer.Kaivers@med.uni-duesseldorf.de (J.K.); Kathrin.nachtkamp@med.uni-duesseldorf.de (K.N.); haem-onk.haas@med.uni-duesseldorf.de (R.H.); Kobbe@med.uni-duesseldorf.de (G.K.); gattermann@med.uni-duesseldorf.de (N.G.); germing@med.uni-duesseldorf.de (U.G.)

**Keywords:** myelodysplastic syndromes, guidelines, patient reported outcome, prognosis

## Abstract

The heterogeneous group of myelodysplastic syndromes (MDS) needs an individualized and patient-tailored therapeutic approach. Consensus-based guidelines for diagnosis and treatment provide a basis for clinical decision making. MDS guidelines are issued by expert panels. Our main objective was to examine how guidelines influence patients’ adherence to expert recommendations and how they ensure healthcare quality. To approach this question, we reviewed the most common guidelines for diagnosing and treating MDS in adult patients. Furthermore, we critically looked at quality indicators for everyday practice and studied adherence in an everyday outpatient setting. Finally, we also paid close attention to patient-reported outcome measures and studied how they are used as endpoints in clinical trials. We can conclude that the combination of evidence-based diagnostic tools, standardized treatment recommendations, and patient-centered shared decision making will eventually lead to a healthcare standard that will significantly improve outcomes in adult patients with MDS.

## 1. Introduction

Myelodysplastic syndromes (MDS) are clonal disorders of hematopoietic stem and progenitor cells mainly affecting the elderly population [1]. The clinical course may vary from mild symptoms of peripheral cytopenia to rapid progression to acute myeloid leukemia (AML), in most cases leading to shorter overall survival (OS) and reduced quality of life (QoL). The heterogeneous nature of MDS demands an individualized therapeutic approach. In recent years, substantial progress in clinical research has helped to understand the aspects that shape the prognosis and outcomes in patients with MDS. Advances that are relevant for everyday practice eventually find their way into consensus-based guidelines. Such guidelines provide a framework for clinical decision making. A perfect guideline enhances healthcare quality by ensuring diagnostic accuracy, promoting therapeutic efficacy, and advising against unnecessary or even harmful interventions.

When analyzing adherence to guidelines, the critical question arises as to how the guidelines were conceived. Methodology is often non-transparent and varies greatly depending on the disease and the institution that organizes the authoring. In addition, the status of approval of pharmaceutical products in different countries must be taken into account when guidelines are established and also when adherence to guidelines is assessed. While there is an abundance of practice guidelines for solid tumors, often based on extensive multicenter phase III clinical trials, MDS guidelines are commonly issued by international expert panels drawing on their clinical expertise. For patients with lower-risk MDS and those who cannot tolerate intensive treatment, therapeutic guidelines generally concentrate on improving the QoL, whereas guideline recommendations for patients with higher-risk MDS are primarily concerned with extending OS, particularly emphasizing the importance of identifying patients who may be eligible for allogeneic stem cell transplantation (alloSCT).

Although the results of clinical trials serve as cornerstones in guideline development, clinical trials designed to prove the efficacy and safety of drugs are not well suited to assess how well patients are going to adhere to a particular treatment once it has been incorporated into the guidelines.

As guideline adherence becomes more widely used for healthcare quality assessment, it is important for clinicians not only to be familiar with guidelines, but also to understand how patients perceive guideline recommendations and why recommended actions may fail despite good intentions. To raise awareness of this topic, we have reviewed the work of experts in the field of guideline adherence and healthcare quality in MDS.

## 2. A Brief Reference to Guideline Adherence in Solid Tumor Treatment

Heins et al. [2] studied adherence to first line treatment recommendations in solid cancer. An expert panel representing seven medical specialties typically involved in clinical decision making for cancer patients reviewed current Dutch treatment guidelines for some of the most common malignancies, namely breast cancer, non-small cell lung cancer, small cell lung cancer, prostate cancer, colorectal cancer, and melanoma. The National Dutch cancer guidelines are available online. For each tumor entity, experts defined typical patient profiles based on their clinical expertise. For their analyses, they selected those patient profiles with clear-cut recommendations for or against a specific treatment.

The national cancer registry of the Netherlands provided information on individual adult patients with cancer, regarding patient-specific features like age and comorbidities, as well as disease-related features like cancer type, initial stage at diagnosis, and treatment given. Patients matching the defined profiles were retrospectively identified among those older than 18 years and diagnosed between 2007 and 2012. The group managed to compile sizeable profile-fitting cohorts ranging from 1,160 patients with lung cancer to about 15,000 patients with breast cancer. The cases identified in the registry were then checked for guideline adherence.

The mean adherence to therapeutic guideline recommendations varied from 40 to 99%, strongly depending on the tumor entity. A high level of adherence was found in melanoma and breast cancer patients (99% each), whereas adherence was much lower in lung cancer patients (53%). Interestingly, recommendations that advised against a treatment had considerably higher rates of adherence than recommendations that recommended a specific treatment (98 vs. 75%, *p* < 0.001). Adherence was also dependent on the type of therapy, ranging from a mean adherence of 44% for recommendations involving chemoradiation to 92% in those focusing on hormonal therapy.

This study in adult patients with solid tumors highlights the considerable variation in adherence to different recommendations made in cancer treatment guidelines. The authors conclude that the observed variance across cancer types and treatment modalities at least suggests that adherence could be further improved. Diagnostic guidelines have not been addressed in this study.

## 3. Guidelines: The Key to Quality Care in MDS

Clinical practice guidelines for diagnosing and treating MDS are issued by national and international working groups. Guidelines by the National Comprehensive Cancer Network (NCCN), which cover solid tumors and hematological malignancies, including MDS [3], are commonly used in the US. European guidelines include the European LeukemiaNet (ELN) recommendations [4], last amended in 2014, and the European Society for Medical Oncology (ESMO) MDS guidelines [5]. National MDS guidelines, like those included in the Onkopedia guidelines in Germany [6] or the Scandinavian practice manuals (NMDS) [7], as well as further national guidelines take into consideration special demographical features as well as the national approval status of drugs.

**Figure 1:**
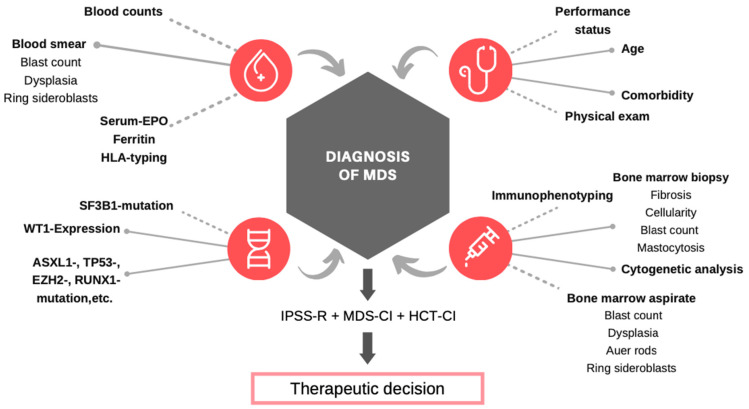
Overview of diagnostic tools for diagnosing MDS.

The diagnosis of MDS may be suggested by a patients’ medical history and a thorough physical examination, confirmed by the microscopic examination of blood and bone marrow specimens, and corroborated by cytogenetics, flow cytometry, and mutation analysis. The complete array of diagnostic techniques may be required to rule out the most important differential diagnoses, i.e., AML and aplastic anemia (AA). Following standardized procedures improves the accuracy and swiftness of diagnosis and allows for the identification of possible therapeutic targets (Figure 1).

The diagnosis of MDS consists of several elements, which together define a standardized diagnostic approach that enables physicians to make an accurate diagnosis and plan adequate treatment. The first element is taking a detailed medical history, paying attention to (1) previous exposure to mutagenic agents like cytotoxic drugs used for chemotherapy, as well as radiotherapy or radioiodine treatment. This information is important to make or to exclude a diagnosis of treatment-related MDS, and also helps to assess the patient’s prognosis. (2) Occupational hazards such as long-term exposure to benzene must be considered, too, as they may justify the recognition of MDS as an occupational disease. (3) Comorbidities must certainly be recorded because they may influence therapeutic decision making. For instance, a history of frequent falls or easy bruising or gastrointestinal bleeding suggests that serious bleeding complications may occur during treatment regimens that induce or aggravate thrombocytopenia. A history of frequent infections may point to an increased risk of septic complications during treatment. Furthermore, the medical history should consider the nutritional status, current medication, prior or concurrent neoplasms, and risk factors for hepatitis and HIV infection.

The second diagnostic element is a thorough physical examination, which can yield more information about cytopenia-related signs and symptoms: dyspnea, tachycardia, petechial bleedings, pleural or pericardial effusion, inflammatory changes, constitutional symptoms, organomegaly, and lymphadenopathy (particularly in chronic myelomonocytic leukemia, CMML). Patients should also be screened for vascular events, again especially in MDS/MPN (myeloproliferative neoplasms) overlap syndromes such as CMML and RARS-T (refractory anemia with ring sideroblasts and thrombocytosis).

The third pillar of MDS diagnosis is the morphological assessment of blood and bone marrow. A complete blood count and a peripheral blood smear to detect dysplasia or other abnormalities are mandatory. It may be useful to assess peripheral blood smears repeatedly at the time of first diagnosis.

A well-performed bone marrow biopsy is the core element of MDS diagnosis. The aspirate should provide enough material for a series of bone marrow smears, including specimens for special cytochemical staining, as well as flow cytometric analysis. Cytomorphological examination of the bone marrow smears should always be guided by a clear clinical question and knowledge of the blood cell counts, in order to enable a meaningful interpretation. If only subtle signs of dysplasia are present in the bone marrow, cytomorphological assessment of peripheral blood cells is important to differentiate between MDS and idiopathic cytopenia of unknown significance (ICUS). As cytomorphological evaluation strongly depends on the experience of the hematologist or pathologist, diagnosis accuracy may be augmented by flow cytometry. In addition, histopathologic examination of a trephine biopsy should be performed in order to achieve a more reliable estimate of bone marrow cellularity and detect the presence of fibrosis.

The fourth element of MDS diagnostics is karyotype and mutation analysis of blood and bone marrow cells. This part is essential, because the results are critical for determining the correct MDS subtype and assessing the patient’s prognosis. The results may also identify anomalies that can be targeted by specific drugs. Chromosomal aberrations are detectable by conventional cytogenetic analysis and fluorescence in-situ hybridization (FISH), while somatic mutations can be detected by polymerase chain reaction (PCR) and next-generation sequencing using targeted gene panels or whole exome analysis.

Cytogenetic and mutation analyses are also relevant for separating MDS from clonal hematopoiesis of indeterminate potential (CHIP) and possibly clonal hematopoiesis of oncogenic potential (CHOP) [8]. Increasing the availability of screening methods for somatic mutations will increasingly produce situations where the distinction between MDS and pre-MDS conditions like ICUS or CCUS is difficult. The use of mutational analysis is certainly recommended by guidelines [9], especially in low-risk MDS, but it is unclear to what extent the guidelines are followed in daily clinical practice. Data published by our group demonstrate that only a decade ago, alarmingly few elderly MDS patients were diagnosed according to the guidelines in Germany, mainly because cytogenetic analysis was omitted [10]. We found that a risk assessment according to the IPSS was less frequently performed in patients over the age of 75 years. Particularly, mutational and cytogenetic analyses were neglected in these patients. Furthermore, age with a cut off of 75 years was found to be a strong predictor for performing a risk assessment in multivariate analyses, as well as a predictor for initiating active treatment. Younger patients were more likely to receive active treatments (i.e., chemotherapy, immunomodulatory therapy, or epigenetic therapy). This work demonstrates that diagnostic guidelines secure a thoroughness of MDS diagnosis and support the decision for a therapeutic option.

A recent study on the same topic showed that diagnostic procedures in the non-academic setting have improved, perhaps because of the availability of licensed medications that can only be used for patients with a narrowly defined MDS subtype [11]. Nevertheless, outside academic centers, there is often no comprehensive, guideline-adherent diagnostic work up for patients with MDS, not even in a rich country like Germany.

## 4. MDS Practice Guideline Recommendations

As our overview illustrates (Table 1), the most commonly used MDS guidelines are quite similar and differ only with regard to the national approval status of drugs. This similarity facilitates the analysis of guideline adherence.

The first step in treating MDS is defining a treatment goal. Treatment goals in patients with MDS are twofold. Improving cytopenias and reducing complications like falls or bleedings is the goal for patients with lower risk MDS. In those patients with higher risk disease, the goal, however, is to alter the natural course of the disease.

Treatment strategies in lower risk patients include regular transfusions, iron chelation to prevent secondary hemochromatosis, antibiotic and antifungal therapy during potential infectious complications, and granulocyte-colony-stimulating factor (G-CSF) administration. This treatment course is called best supportive care, and ought to be the cornerstone in treating MDS patients so as to reduce the burden of disease.

Furthermore, erythropoiesis-stimulating agents (ESA), such as high dose erythropoietin (EPO), play a key role in patients with symptomatic anemia needing regular transfusions. Patients with a low transfusion burden of less than two units per months and an EPO level of less than 500 U/l are expected to have a superior response rate than those with a higher endogenous EPO level [12].

Patients with hypocellular bone marrow are eligible for immunosuppressive approaches with antithymocyte globulin or cyclosporine. Both treatments are used to treat AA and have also been proven to be effective in this subgroup of MDS patients.

Another special subgroup is represented by patients with del(5q). Bone marrow analyses often reveal ring sideroblasts paired with a SF3B1 and/or JAK2-mutation. Responses to ESA were often lower than in non-del(5q) patients, and hence they are eligible for treatment with Lenalidomide [13]. Lenalidomide is approved in the United States for the treatment of patients with del(5q) MDS with low- and intermediate-risk according to the IPSS. This group may include patients with del(5q) plus one other chromosomal abnormality or very rare patients with isolated anemia who have two or more additional chromosomal aberrations. In Europe, approval has been granted for patients with IPSS one low- or intermediate-risk and isolated del(5q) only.

Higher risk patients are eligible for therapy with hypomethylating agents (HMAs). Azacytidine is approved for higher risk patients in Europe and for patients of all risk groups in the United States. MDS patients exhibit genome-wide hypomethylation and CpG island hypermethylation, which results in genetic instability typical of cancer and tumor suppressor gene silencing. Hypomethylating agents help to erase the aberrant methylation, resulting in the activation of tumor suppressor genes.

As alloSCT is the only curative treatment eligible, high-risk patients should be offered a consultation to discuss alloSCT close to the time of their initial diagnosis.

What are the core values of an MDS guideline? Which components are important enough to be considered useful indicators of the quality of care?

Stojkov et al. [14] from the Swiss MDS working group asked a panel of 29 MDS experts to define quality indicators for MDS patient care based on 11 relevant guidelines published by national and international working groups since 1999. Following a Delphi procedure, 29 guideline-based indicators (GBIs) from three main domains (diagnosis, therapy and provider/infrastructural characteristics) were identified.

The diagnostic domain includes aspects related to diagnostic procedures, risk stratification using risk scores like the IPSS-R, and patient-reported outcomes. The therapeutic domain emphasizes supportive care as a basic concept for all patients. Further treatment options are divided into those most relevant for lower-risk MDS, i.e., erythropoiesis stimulating agents and Lenalidomide, and those more relevant for higher-risk MDS, i.e., hypomethylating agents and alloSCT. Infrastructural features include a multidisciplinary care team and access to clinical trials.

The selected quality indicators are measurable and can be used to establish improved process quality in MDS patient care. GBIs should be representative of the cornerstones of clinical decision making. Nevertheless, there is a potential pitfall: although it is important to consider GBIs during the diagnosis and treatment of MDS, we should keep in mind that clinical decision making is a process shared between the physician and patient. Patient-related factors and patient preferences play a key role. Thus, we further examined adherence to MDS guidelines in a real-world setting.

## 5. Therapeutic Recommendations: A Bridge between Evidence and Action

Our MDS working group in Düsseldorf studied adherence to treatment recommendations based on ELN guidelines. We conducted a prospective study including 381 adult patients with MDS who received an ELN-based treatment recommendation in our outpatient clinic [15]. During an observation period of four years (2015–2019), we evaluated whether patients followed these recommendations or preferred to choose an alternative approach. Furthermore, we compared the outcomes of adherent versus non-adherent patients. Our primary endpoint was overall survival. The adherence rate was 67%. Adherence did not correlate with the type of MDS. The highest level of adherence was found with regard to best supportive care recommendations, including RBC transfusions and hematopoietic growth factors, as well as allogeneic hematopoietic stem cell transplantation (alloSCT). Low levels of adherence were found for recommendations regarding watchful waiting and the use of Lenalidomide.

Considering the limited number of therapeutic options available, an overall adherence of 67% appeared rather low. Is this detrimental to the outcome? We were curious to know whether guideline adherence really has an impact on the prognosis of MDS patients. Therefore, we performed a retrospective analysis of 1681 patients, comparing those who were eligible for a certain treatment and accepted it, and those who chose not to adhere to the treatment recommendation. The results were disappointing: only patients who adhered to a recommendation for alloSCT or iron chelation gained a significant survival benefit over those who did not follow such recommendations. Adherence to other treatment recommendations did not result in a survival advantage.

Our findings do not invalidate the ELN guideline that we looked at nor any other guideline presented in Table 1. They rather emphasize that proper patient management should go beyond guidelines and should involve shared decision making. Guidelines should be considered a useful framework rather than a dogma. Unfortunately, we were often unable to ascertain the specific reasons for non-adherence to the guideline, mainly because many patients were managed outside our tertiary referral center.

Several factors influence adherence in everyday practice. In general, professionals are more likely to follow recommendations if they are part of a set of guidelines they are familiar with [16]. Furthermore, guidelines cannot be applied to every patient. Individual patient characteristics like performance status, social network, and personal preferences are pivotal in clinical decision making. Our study merely assessed the level of adherence and its influence on overall survival, without taking into account patient-reported outcomes related to quality of life. As QoL is an important component of therapeutic success, it should not be ignored when analyzing the impact of guideline adherence.

## 6. Patient Reported Outcomes: Maximizing Therapeutic Success

Studies on the treatment outcome in MDS patients usually focus on OS and transformation into AML as endpoints, because measuring them is easy. However, patient-reported outcome measures (PROMs) should be included as well. PROMs include information about patients’ general health condition, burden of disease, and other aspects influencing health-related quality of life [17]. Impairments captured by PROMs may provide independent prognostic information [18]. Compared with OS and AML transformation, PROMs are harder to assess and are also subject to considerable inter-observer variability.

Stauder et al. [19] conducted a large meta-analysis, reviewing 275 scientific articles and drug labelling claims since 2000, in order to evaluate the exploitation of patient-reported outcome measures. The group identified 112 PROMs used in MDS patients. Only five were MDS-specific. Among the 152 PROMs used in the AML patients, nine were AML-specific. PROMs covered symptom burden, overall quality of life, and more specific physical and psychological features. Interestingly, domains like coping or resilience were less likely to be measured. Instruments frequently employed were questionnaires like the EORTC Core Quality of life Questionnaire (EORTC QLQ-C30) [20], which was used in more than a third of the reviewed studies. A more symptom-specific questionnaire is the Functional Assessment of Chronic Illness Therapy-Fatigue (FACIT-Fatigue) [21], used in MDS and AML.

In clinical trials, PROMs were mainly used as secondary end points, and more frequently employed in phase 3 than phase 1 or 2 studies. The focus was mainly on health-related quality of life, assessing symptom burden, and physical and psychological features. Only 3% of the trials used PROMs as a primary endpoint.

Stauder et al. also studied whether PROMs were included in labelling claims. They searched the PROLABELS database to identify EMA- and FDA-approved drugs for the treatment of MDS and AML. In total, 13 drugs were identified, of which only two, namely lenalidomide and azacytidine, included PROMs in their labelling claims for MDS treatment submitted to the EMA. PROMs are only used as secondary endpoints. In contrast with the EMA, the FDA does not include PROMs in their MDS drug approvals. Among all of the FDA- or EMA-approved drugs approved for the treatment of AML, none report PROM data in their labels.

We must be aware that, despite guideline recommendations and physician expertise, it is the patient who, consciously or unconsciously, makes the really important decisions. The patient seeks advice from a trusted physician, gives consent to certain diagnostic procedures, and decides which therapeutic recommendations to follow. It cannot be the intention of guidelines to put pressure on patients. Instead, guidelines should facilitate clinical decision making by offering and ranking therapeutic options that are corroborated by evidence from clinical trials or, at least, expert consensus. We should like to emphasize that there is no obligation to use a certain drug simply because it is formally approved for the patient’s medical condition. Guideline-adherent treatment must be aligned with the moral principle of primum non nocere. In other words, rather than simply following guidelines, physicians must take into consideration that a guideline recommendation may actually harm the patient. Guideline adherence is not an end in itself.

## 7. Future Direction: Taking Action

Guidelines for the diagnosis and treatment of MDS try to augment healthcare quality for MDS patients. We considered the physicians’ perspective by looking at guideline-based core indicators reflecting diagnostic workup and therapeutic options [14]. Furthermore, we analyzed adherence in an everyday outpatient setting [16]. Finally, we also paid attention to patient-reported outcome measures and studied how they are used as endpoints in clinical trials [19].

We can show (Table 1) that various MDS guidelines are in fact quite similar and thus cannot be the cause for large differences in guideline adherence. Instead, adherence depends on numerous other factors.

From the sparse literature, we conclude that more needs to be done. Research and scientific evidence in MDS are evolving rapidly, especially in the field of molecular and immunological findings. Guidelines are thus frequently adapted. However, in our view, more emphasis should be placed on PROMs in order to be better improve the quality of care.

In closing, we would like to mention a project that is designed to merge all important diagnostic and therapeutic aspects under the slogan “providing the right care to the right patient with MDS at the right time”. The MDS-RIGHT project is designed for patients with lower-risk MDS and symptomatic anemia. Fifteen European partner organizations joined forces to implement standardized diagnostic and therapeutic interventions in order to promote more effective use of healthcare resources and boost treatment compliance [22]. The project was supported by the European Union between 2015 and 2020. The participants concluded that the combination of evidence-based diagnostic tools, standardized treatment recommendations, and patient-centered shared decision making will lead to a healthcare standard that will significantly improve patients’ outcomes.

Our work emphasizes that proper patient management should go beyond guidelines and should involve shared decision-making. Guidelines should be considered a useful framework rather than a dogma.

## Figures and Tables

**Table 1:** Overview of national and international MDS practice guideline recommendations.

Guidelines and Year Published	Diagnostic Work up	Therapeutic Options	
	mandatory	Lower risk (IPSS low and intermediate 1)	Higher risk (IPSS intermediate 2 and high)
European Leukemia Net (ELN), 2014 [4]	Peripheral blood smear Bone marrow aspirate Bone marrow biopsy Cytogenetic analysis FISH Flow cytometry Immunophenotyping	Baseline Red blood cell (RBC) transfusion and iron chelation if serum Erythropoietin (sEPO) <500 mU/mL and/or RBC transfusion <2 U/month → EPO + G-SCF if del(5q) + sEPO >500 mU/mL and RBC transfusion ≥2 U/month → Lenalidomide if Age <60 years, BM blasts <5%, normal cytogenetics, transfusion-dependency (hypocellular bone marrow) → anti-thymocyte globulin (ATG) + cyclosporin A (CSA)	Age >65–70 years or poor performance status → Azacytidine <65–70 years and good performance status and available stem cell donor → allogeneic stem cell transplantation (alloSCT)
National Comprehensive Cancer Network (NCCN), 2020 [3]	Peripheral blood smear Bone marrow aspirate Bone marrow biopsy Cytogenetic analysis FISH Flow cytometry Immunophenotyping	Baseline RBC transfusions and iron chelation If MDS del(5q) → Lenalidomide If Serum EPO ≤500 mU/mL → Epoetin alfa ± G-CSF or Darbepoetin alfa ± G-CSF If Serum EPO >500 mU/mL → ATG +/− CSA Symptomatic multilineage cytopenia → Azacytidine/Decitabine	Transplant candidate and stem cell donor available → alloSCT or Azacytidine followed by SCT or Decitabine followed by SCT or High-intensity chemotherapy followed by SCT No transplant candidate or no donor available → Azacytidine or Decitabine or clinical trial
German Society of Hematology and Oncology (DGHO), 2021 [6]	Peripheral blood smear Bone marrow aspirate Bone marrow biopsy Cytogenetic analysis FISH Flow cytometry Immunophenotyping	Baseline RBC transfusions and iron chelation If MDS-RS/SF3B1+/− sEPO > 500 U/L or EPO refractory → Luspatercept If MDS del(5q) → Lenalidomide If sEPO < 500 U/l or 2 RBCs/month → EPO If Age <60 years, BM blasts <5%, normal cytogenetics, transfusion-dependency (hypocellular bone marrow) → ATG + CSA	Age >65–70 years or poor performance status → BSC and Azacytidine <65–70 years and good performance status and available stem cell donor → alloSCT
ESMO, 2020 [5]	Peripheral blood smear Bone marrow aspirate Bone marrow biopsy Cytogenetic analysis FISH Flow cytometry Immunophenotyping	Baseline RBC transfusions and iron chelation If MDS del(5q) → Lenalidomide If RBC transfusions ≥2 concentrates/month and serum EPO ≥500 U/l → EPO (if no MDS del(5q)) If RBC transfusions <2 concentrates/month and/or serum EPO <500 U/l → EPO + G-CSF	Fit patients of ≤70 years with a donor for alloSCT → alloSCT Patients of >70 years or younger but without a donor for alloSCT → Azacytidine

## Data Availability

Not applicable.
